# Extended Pharmacokinetic Model of the Intravitreal Injections of Macromolecules in Rabbits. Part 2: Parameter Estimation Based on Concentration Dynamics in the Vitreous, Retina, and Aqueous Humor

**DOI:** 10.1007/s11095-020-02946-1

**Published:** 2020-10-22

**Authors:** Marko Lamminsalo, Timo Karvinen, Astrid Subrizi, Arto Urtti, Veli-Pekka Ranta

**Affiliations:** 1 School of Pharmacy, Faculty of Health Sciences, University of Eastern Finland, P.O. Box 1627, FI-70210 Kuopio, Finland; 2 Granlund Consulting Oy, Helsinki, Finland; 3 Division of Pharmaceutical Biosciences, Faculty of Pharmacy, University of Helsinki, Helsinki, Finland; 4 Laboratory of Biohybrid Technologies, Institute of Chemistry, St. Petersburg State University, St. Petersburg, Russian Federation

**Keywords:** computational fluid dynamics, intravitreal injection, macromolecule, Ocular pharmacokinetics, permeability

## Abstract

**Purpose:**

To estimate the diffusion coefficients of an IgG antibody (150 kDa) and its antigen-binding fragment (Fab; 50 kDa) in the neural retina (D_ret_) and the combined retinal pigment epithelium-choroid (D_RPE-cho_) with a 3-dimensional (3D) ocular pharmacokinetic (PK) model of the rabbit eye.

**Methods:**

Vitreous, retina, and aqueous humor concentrations of IgG and Fab after intravitreal injection in rabbits were taken from Gadkar et al. (2015). A least-squares method was used to estimate D_ret_ and D_RPE-cho_ with the 3D finite element model where mass transport was defined with diffusion and convection. Different intraocular pressures (IOP), initial distribution volumes (V_init_), and neural retina/vitreous partition coefficients (K_ret/vit_) were tested. Sensitivity analysis was performed for the final model.

**Results:**

With the final IgG model (IOP 10.1 Torr, V_init_ 400 μl, K_ret/vit_ 0.5), the estimated D_ret_ and D_RPE-cho_ were 36.8 × 10^−9^ cm^2^s^−1^ and 4.11 × 10^−9^ cm^2^s^−1^, respectively, and 76% of the dose was eliminated via the anterior chamber. Modeling of Fab revealed that a physiological model parameter “aqueous humor formation rate” sets constraints that need to be considered in the parameter estimation.

**Conclusions:**

This study extends the use of 3D ocular PK models for parameter estimation using simultaneously macromolecule concentrations in three ocular tissues.

**Electronic supplementary material:**

The online version of this article (10.1007/s11095-020-02946-1) contains supplementary material, which is available to authorized users.

## Introduction

Blood-ocular barriers protect the eye and pose a major challenge in the treatment of posterior segment diseases, such as age-related macular degeneration (AMD) (1). Reaching the drug targets in the retina requires effective drug delivery techniques (2,3), and in the case of AMD, therapeutic levels of anti-VEGF proteins such as bevacizumab, ranibizumab, and aflibercept in the retina can only be achieved via intravitreal (IVT) injection (4–7). However, IVT injections are invasive, costly and need to be repeated monthly or bimonthly (6,8). Longer acting and retina-targeting dosage forms are an important goal in current retinal drug development (9,10).

The ocular half-life of biologicals in humans is typically 5–10 days and about half of that in rabbits (11,12). The elimination of biologicals after IVT injection takes place anteriorly via aqueous humor (AH) outflow and posteriorly across the blood-retina barrier. Contradictory statements on the importance of these routes have appeared in the literature as discussed in a recent review (3). The classical model by David Maurice and more recent modeling studies on macromolecules showed that the anterior route is dominating in rabbits. This conclusion was based on the finding that the models were able to explain the observed ratio of aqueous humor (AH) concentration to vitreous concentration (13–15) or the complete concentration curves in the vitreous, retina, and AH (16). Additionally, Araie and Maurice (17) obtained experimental verification for the dominance of the anterior route by comparing the concentration contours of fluorescein isothiocyanate dextran (66 kDa) with those of fluorescein in rabbit eyes that were frozen after the diffusional equilibrium had been reached.

Traditional compartmental pharmacokinetic (PK) models have been used to describe ocular drug concentration profiles and estimate PK parameters, such as clearance, apparent volume of distribution, elimination half-life, and permeability (11,16,18,19). However, compartmental models assume homogenous drug concentration in each ocular tissue which is not realistic, especially in the vitreous. This deficiency has been remedied with the finite element modeling (FEM) which is based on anatomically accurate three-dimensional (3D) geometric models consisting of thousands of tiny compartments to simulate localized drug concentration profiles in hard-to-reach ocular tissues (15,20–23). These models incorporate physical phenomena, such as diffusion, convection, and heat transfer, and molecular characteristics, such as diffusion coefficient, and permeability.

Several 3D ocular FEM models have been used to understand and predict macromolecule concentration profiles in the retinal drug delivery. These models have afforded new insights into the different delivery routes and mixing in the vitreous, the concentration profiles in several ocular tissues (AH, vitreous, retina) and species (rabbits, humans and monkeys) after IVT injection (20,21,24).

An important part of PK modeling is parameter estimation using measured drug concentrations, but ocular FEM models have been used sparsely for this purpose. Haghjou et al. (25) estimated the combined retina-choroid-sclera permeability for 32 drugs after IVT injection with a least squares method using drug concentrations in the vitreous. Recently, Zhang et al. (21) estimated clearance parameters for bevacizumab, ranibizumab and sodium fluorescein after IVT and suprachoroidal injection by simulating drug concentration profiles with several parameter values (a grid search). These models are restricted to the posterior segment of the eye and do not describe realistically the elimination of macromolecules through the anterior pathway. FEM models of the complete eye have not been used earlier for parameter estimation.

The general aim of our study was to extend our previously published model (15) for parameter estimation. The specific objective was to estimate the diffusion coefficients of IgG antibody (150 kDa) and its antigen-binding fragment (Fab; 50 kDa) in the neural retina (D_ret_) and the combined retinal pigment epithelium-choroid (D_RPE-cho_) of the rabbit eye. Using vitreous, retina and AH concentration data of IgG and Fab after IVT injection in rabbits (26), a formal least-squares method was used to estimate D_ret_ and D_RPE-cho_ based on all concentration data for each macromolecule. Earlier, Hutton-Smith et al. (16) used the same data to estimate inner limiting membrane (ILM) and RPE permeabilities for IgG and Fab using a semi-mechanistic model with three well-stirred compartments, but an additional virtual delay chamber was needed to move the peak concentration in AH from time zero to the correct time. Our model uses an anatomically accurate geometry with FEM approach which dismisses the need for a non-physiological delay-compartment and provides a novel tool for quantitative ocular barrier analysis.

## Materials and Methods

The methodology is based on our earlier model (15) that was an extension of the model originally developed by Missel (20). This section gives a general description of the previously published methods and a detailed description of new features. Detailed information on methods is given in the Electronic Supplementary Material.

### Software and the Base Model

The finite element modeling (FEM) was carried out using COMSOL Multiphysics 5.4 software (COMSOL AB, Stockholm, Sweden). The model with heat transfer and gravity (enhanced mixing in the anterior chamber) from our earlier study (15) was built with the following modules: laminar flow, heat transfer in fluids, non-isothermal flow multiphysics, and transport of diluted species. It was extended to parameter estimation using optimization module. The 2D-axisymmetric geometry of rabbit’s eye without the canal of Petit from our earlier study (15) was used. The geometry was divided into 36,081 elements using COMSOL’s predefined physics-controlled mesh sequence with extra fine element size. Simulations were run with Intel Core i7–6700 16 GB RAM PC running under Windows 10.

Incompressible flow of water with Navier-Stokes equations in free flow and Brinkman equations in porous media were used for AH flow. Heat transfer from the posterior eye towards the cornea was defined using a convection-diffusion equation for heat conservation. Gravity was set anterior-to-posteriorly along the symmetry axis. Mass transfer in the non-isothermal flow was restricted to vitreous, neural retina, RPE-choroid, anterior and posterior chamber and trabecular meshwork. All other domains were impermeable to macromolecules with no slip boundary condition. Detailed equations are presented in Electronic Supplementary Material (Chapters 2 and 3).

### In Vivo Data

Experimental data were taken from a comprehensive ocular PK study on human antiglycoprotein D derived IgG antibody (IgG) and its antigen-binding fragment (Fab) in rabbits by Gadkar et al. (26). Male New Zealand White rabbits (weight 2.5–3.5 kg) were used in the study (*n* = 12–18 per group). The IVT injection was administered as a single bilateral injection of 500 μg IgG or Fab in 50 μl volume into the inferior vitreous body. Ocular tissues were terminally collected at 6 h, and 2, 7, 14, 21 and 28 days post dosing. Basing on the study of Gadkar et al. (26), our study uses the doses and concentration-time profile data of IgG and Fab in the retina, vitreous and AH explicitly provided by Hutton-Smith et al. (16) (Table S1 in Electronic Supplementary Material). The doses given by Hutton-Smith et al. (16) were 549 μg (3.66 nmol) for IgG and 615 μg (12.3 nmol) for Fab, respectively.

### Initial Distribution Volume of Dose and Intraocular Pressure

**Fig. 1:**
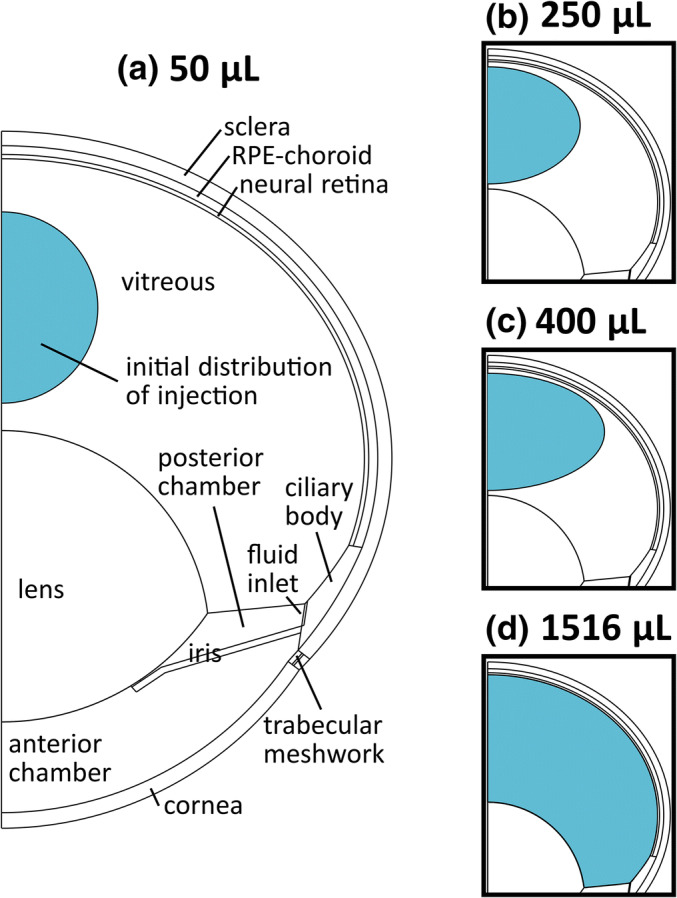
The 2D-axisymmetric geometry of the rabbit eye and the different initial distributions after intravitreal injection (**a**)-(**d**). Note that (**b**)-(**d**) depicts the apparent spread of the IVT injection immediately after the administration, not the actual volume of injection (50 μl). A symmetric 3D eye was obtained by rotating the geometry around the anterior-to-posterior axis

Based on an earlier idea by Zhang et al. (21), the effect of the apparent initial distribution volume of the dose was investigated by varying the apparent spread of the dose in the vitreous immediately after administration. The tested distribution volumes were 50, 250, 400 and 1516 μl, the last representing the total volume of the vitreous (Fig. 1). The exact coordinates and radii of the initial distribution spheres are given in Table S3 of Electronic Supplementary Material.

Intraocular pressures (IOP) of 10.1, 12.5, 15, 17.5 and 20 Torr were used in the simulations. The desired IOP was produced by adjusting the hydraulic permeability of the trabecular meshwork as described in detail in our earlier study (15). In the model, the IOP controls the distribution of AH flow from the inlet port in the ciliary body (influx 3 μL min^−1^) to the outlet ports in a) the trabecular meshwork (TM), b) the whole corneal surface, and c) the whole retinal surface. For the main outlet port, the AH outflow through the TM ranged from 2.917 μL min^−1^ (10.1 Torr) to 2.755 μL min^−1^ (20 Torr) (Chapter 5 in Electronic Supplementary Material).

### Transport of Macromolecules Via Convection and Diffusion

The transport of macromolecules was defined with convection and diffusion as presented earlier (15). Convection was governed by AH flow pattern. Diffusion coefficients of IgG (150 kDa) and Fab (50 kDa) in water (D_wat_) at 37°C were used in AH, TM and vitreous (98% of vitreous is water), and they were calculated with the Einstein-Stokes equation

1 $$ {D}_{wat}=\frac{k_BT}{6\pi \eta {r}_H} $$

where k_B_ is the Boltzman constant (1.381 10^−23^ J K^−1^), T is the absolute temperature (310.15 K), η the dynamic viscosity (0.00069 kg m^−1^ s^−1^) and r_H_ the hydrodynamic radius of the molecule (m) (27). Using r_H_ of 4.9 nm for IgG (28) D_wat_ of 6.73 × 10^−7^ cm^2^s^−1^ is obtained. Similarly for Fab (r_H_ = 2.5 nm; 28), D_wat_ is 13.2 × 10^−7^ cm^2^s^−1^. The transport of macromolecules in anterior and posterior chambers was governed by convection while diffusion dominated in the vitreous (Electronic Supplementary Material, Chapter 6).

**Fig. 2:**
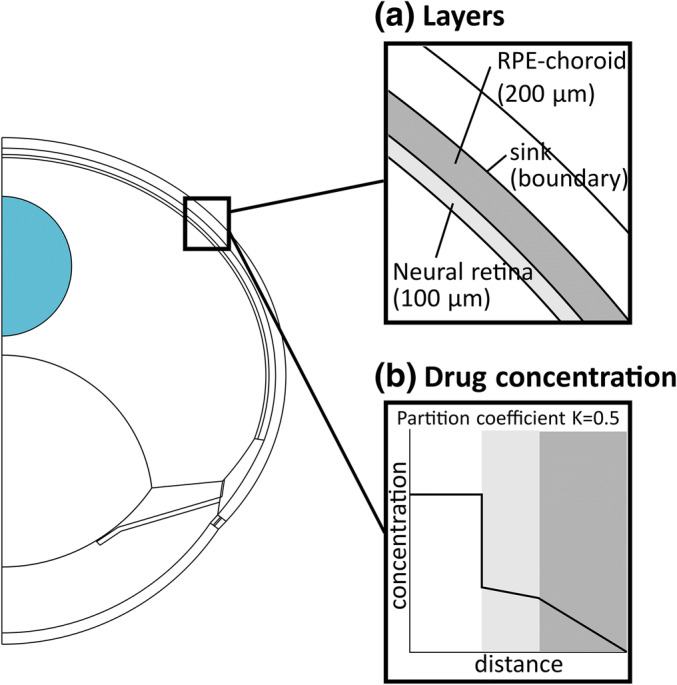
The modified geometry including the neural retina and combined RPE-choroid as separate layers (**a**) and the effect of neural retina/vitreous partition coefficient (K_ret/vit_ = 0.5) at the retina-vitreous boundary on the concentration profile (**b**)

A new feature was to include the neural retina and the combined RPE-choroid as separate domains to formally estimate diffusion coefficients of macromolecules in these layers: D_ret_ and D_RPE-cho_ (Fig. 2a). The combined RPE-choroid was used to avoid mathematical modeling problems in thin RPE. This decision was justified since macromolecule concentrations in the RPE and choroid were not available, and these tissues acted only as an elimination route. Diffusion and convection carried macromolecules in vitreous, but their transport in the neural retina and the RPE-choroid was governed by pure diffusion which was obtained by setting the convection term in these layers to zero. This method leads to realistic simulations as shown previously (15). The sink with zero concentration was at the anterior surface of the sclera.

Another new feature in our model was to include a neural retina/vitreous partition coefficient (K_ret/vit_) at the boundary of these tissues (Fig. 2b). The experimental ratio of mean retinal concentration to mean vitreal concentration of IgG between 3 and 29 days was 0.47 (26, Table S1 in Electronic Supplementary Material). Therefore, K_ret/vit_ of IgG at the exact boundary was set to 0.5, a slightly higher value than the ratio of mean concentrations, to enable a declining concentration profile in the neural retina:

2 $$ {K}_{ret/ vit}\  for\  IgG=\frac{C_{ret, ant}}{C_{vit, post}}=0.5 $$

where C_ret,ant_ is the IgG concentration in the anterior surface of the retina and C_vit,post_ is the IgG concentration in the posterior surface of the vitreous. Based on experimental Fab data by Gadkar et al. (26) (Table S1 in Electronic Supplementary Material), K_ret/vit_ of 0.55 was introduced for Fab. K_ret/vit_ was implemented in COMSOL with a partition condition node in the transport of diluted species physics. The partition coefficient between the neural retina and the combined RPE-choroid was set to 1 for IgG and Fab, since concentration data in RPE and choroid were not available.

### Least Squares Method for the Estimation of Diffusion Coefficients

Parameters D_ret_ and D_RPE-cho_ were estimated by minimizing the objective function value (OFV) with the least squares method using simultaneously vitreous, retina and AH concentrations:

3 $$ OFV=\sum W\times {\left({C}_{calc,t}-{C}_{obs,t}\right)}^2 $$

where W is the weight for the domain, C_calc,t_ the calculated mean concentration and C_obs,t_ the measured mean concentration in the corresponding domain at each time point. In this text, the global sum of squared residuals (global SSR) means the total combined SSR in vitreous, retina and AH. Optimization module in COMSOL was used in parameter estimation. Vitreous, retina and AH were given equal weight each (0.333/0.333/0.333) unless otherwise noted. Derivative-free Bound Optimization by Quadratic Approximation (BOBYQA) solver was used with optimality tolerance of 0.01.

For parameter estimation, initial estimates of D_ret_ and D_RPE-cho_ were calculated for IgG and Fab based on the apparent permeability values by Hutton-Smith et al. (16). The apparent permeability P_app_ is defined as (4) and rearranged in (5) to get D:

4 $$ {P}_{\mathrm{a} pp}=\frac{D\times K}{h} $$

5 $$ D=\frac{P_{app}\times h}{K} $$

where D is the diffusion coefficient (cm^2^s^−1^), K the membrane/water partition coefficient and h the thickness of the layer (100 μm for neural retina and 200 μm for RPE-choroid in this model).

The permeability estimate for the inner limiting membrane (P_ILM_) by Hutton-Smith et al. (16) was used for D_ret_, and the permeability estimate for the RPE (P_RPE_) for D_RPE-cho_, respectively. For IgG, eqs. (2) and (5) combined with permeability estimates (P_ILM_ = 1.7 × 10^−7^ cm s^−1^; P_RPE_ = 1.84 × 10^−7^ cm s^−1^) gave initial D_ret_ of 3.4 × 10^−9^ cm^2^s^−1^ and D_RPE-cho_ of 3.68 × 10^−9^ cm^2^s^−1^, respectively. For Fab (P_ILM_ = 1.88 × 10^−7^ cm s^−1^; P_RPE_ = 2.60 × 10^−7^ cm s^−1^), initial estimates for D_ret_ and D_RPE-cho_ were 3.76 × 10^−9^ cm^2^s^−1^ and 5.20 × 10^−9^ cm^2^s^−1^, respectively.

### Optimization of the Full Model and Sensitivity Analysis

IgG model was optimized by testing all the combinations of IOP (10.1, 12.5, 15, 17.5, and 20 Torr) and the initial distribution volumes (50, 250, 400, and 1516 μl). The final model was chosen by visual inspection of concentration curves and by comparing both global SSR and the calculated versus observed area under the curve (AUC) in each domain. Finally, sensitivity analysis for the most important model parameters was performed. Each parameter value was individually changed to 50% and 200% of that in the final model, and sensitivity was evaluated based on the changes in AUC values and the percentage of dose eliminated via the anterior chamber, the latter calculated with COMSOL’s integration feature. The length of the simulation for IVT injection was 700 h. Each run with the estimation of D_ret_ and D_RPE-cho_ typically lasted 1 h.

## Results

### IVT Injection of IgG

#### Parameter Estimation and Optimization

**Fig. 3:**
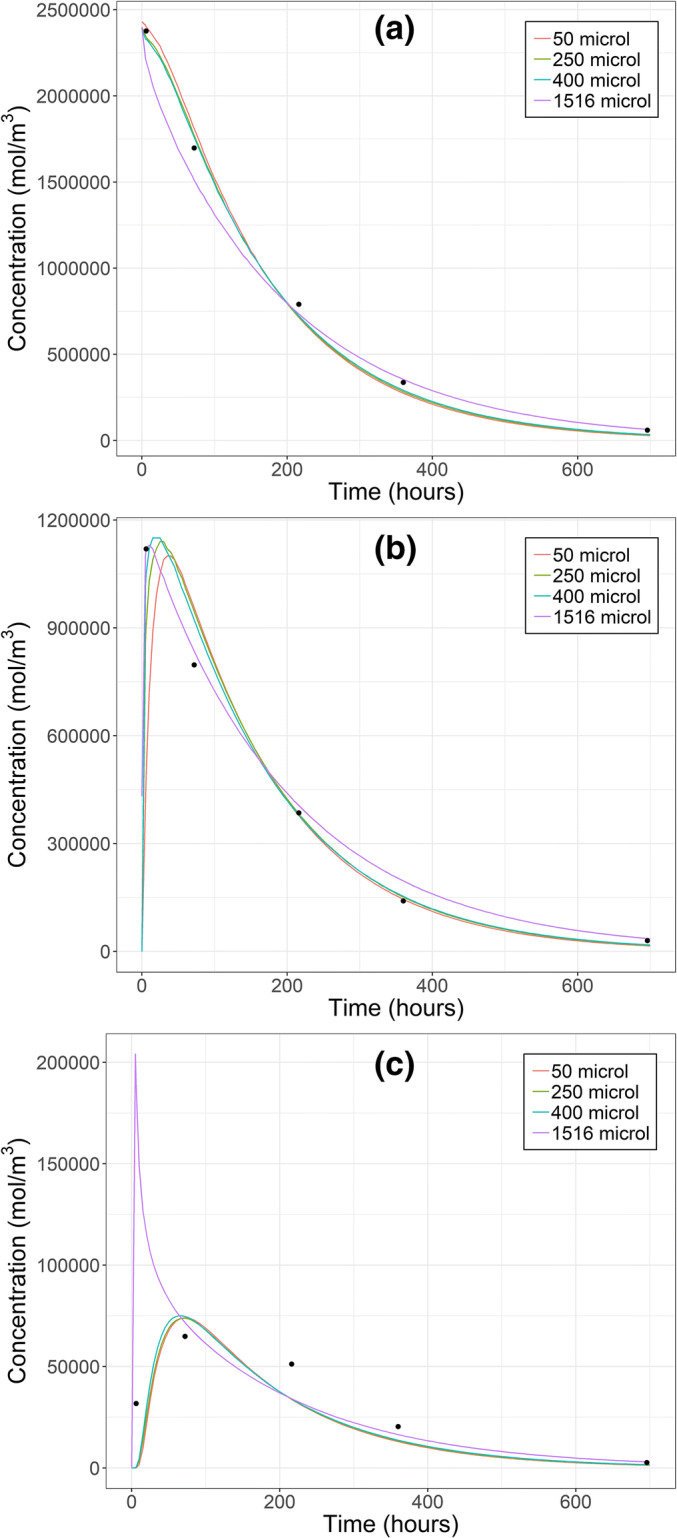
Calculated IgG concentrations after intravitreal injection in vitreous (**a**), retina (**b**) and aqueous humor (**c**) with different initial distribution volumes (lines) and measured concentrations (black dots, 26). Intraocular pressure was 10.1 Torr. Note the different concentration scales in each panel

**Fig. 4:**
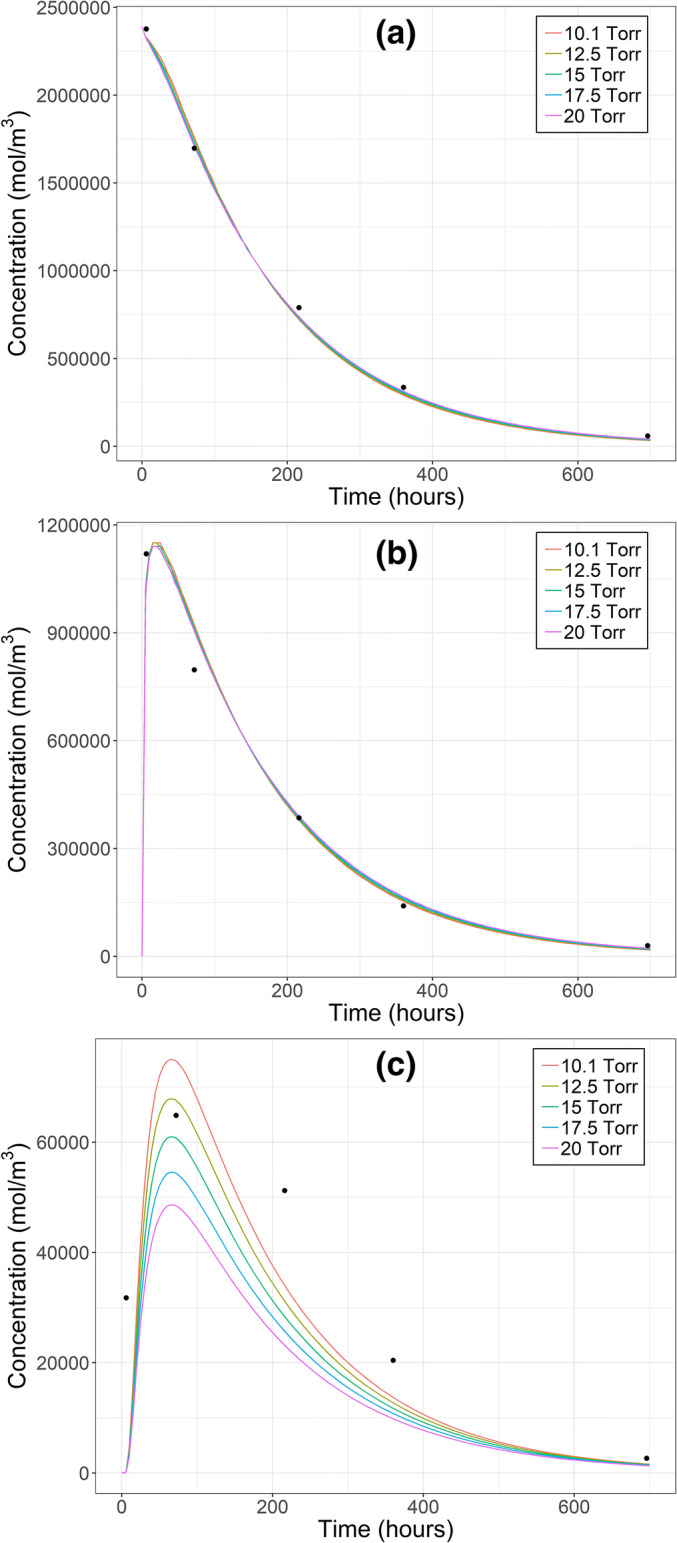
Calculated IgG concentrations after intravitreal injection in vitreous (**a**), retina (**b**) and aqueous humor (**c**) with different intraocular pressures (lines) and measured concentrations (black dots, 26). The initial distribution volume was 400 μl. Note the different concentration scales in each panel

The general trend in IgG modeling and the path to the final model are shown in Figs. 3 and 4. The complete listing of parameter estimates is given in Electronic Supplementary Material (Chapter 7).

At 10.1 Torr the initial distribution volume of 400 μl yielded the best match between the calculated and measured IgG concentrations in vitreous, retina and AH (Fig. 3). While the fits were visually similar at 400 μl and 250 μl, global SSR at 400 μl was only 42% of that at 250 μl. With the actual injection volume (50 μl), the transport of drug from vitreous to retina took too much time resulting in poor fit of retinal concentration in the first time point. On the other hand, with the whole vitreous spread (1516 μl) AH concentration rose rapidly to too high levels. In most cases, the calculated concentrations obtained with different initial volumes were similar starting from 72 h. When the initial volume was between 50 and 400 μl, estimated D_ret_ remained within 6.1-fold range and D_RPE-cho_ within 1.2-fold range, respectively (Electronic Supplementary Material, Chapter 7).

When 400 μl initial distribution volume was tested at all IOPs, marked differences were observed only in AH concentrations (Fig. 4). The best fit was obtained at 10.1 Torr based on better visual match of AH concentrations, and this model was considered as the final model with parameter estimates of 36.8 × 10^−9^ cm^2^ s^−1^ and 4.11 × 10^−9^ cm^2^ s^−1^ for D_ret_ and D_RPE-cho_, respectively. The effect of IOP on D_ret_ and D_RPE-cho_ estimates is discussed in Electronic Supplementary Material (Chapter 9).

**Fig. 5:**
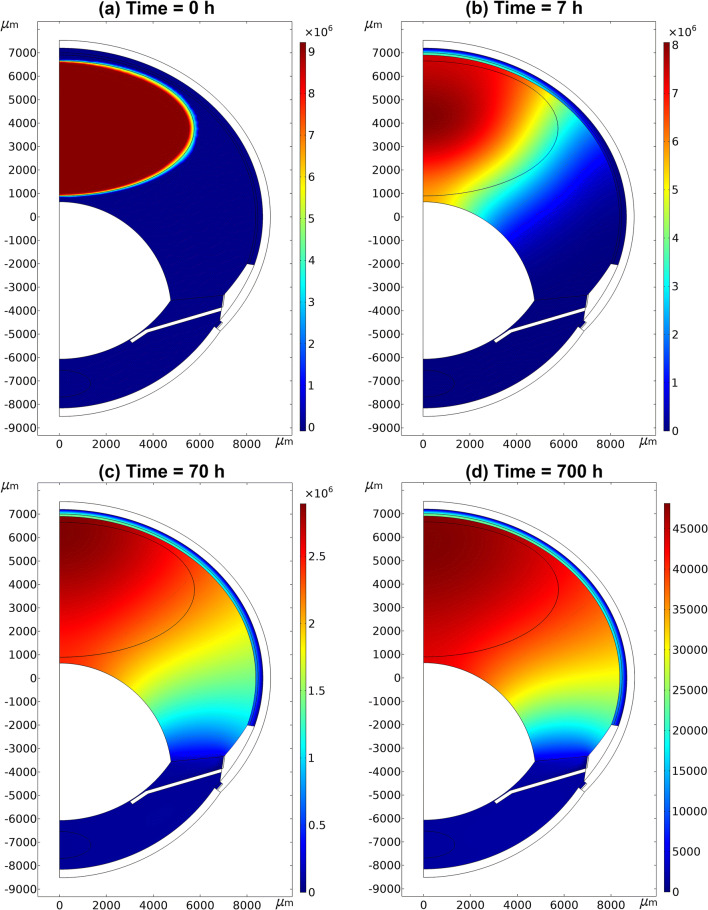
Simulated IgG concentration contours (mol/m^3^) after intravitreal injection with the final model at 0 (**a**), 7 (**b**), 70 (**c**) and 700 h (**d**). The initial distribution volume was 400 μl and intraocular pressure 10.1 Torr. Note the different concentration scales in each panel

**Fig. 6:**
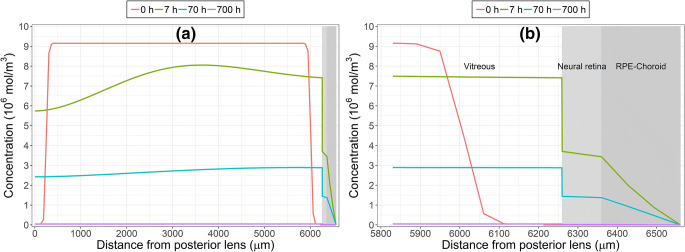
Simulated IgG concentrations on the symmetry axis from the posterior lens (0 μm) to the sink at the anterior sclera (6560 μm) as whole (**a**) and enlarged for the posterior eye (**b**) obtained with the final model. The effect of the neural retina/vitreous partition coefficient (0.5) can be observed at the vitreous-retina boundary at a distance of 6260 μm

Figure 5 illustrates IgG concentration contours of the final model (400 μl, 10.1 Torr) at 0, 7, 70 and 700 h, while Fig. 6 shows the concentration on the symmetry axis at the same time points. The percentages of anterior and posterior (via retina and RPE-choroid) elimination pathways were 76% and 23%, respectively, yielding mass balance of 99% of the dose at the end of the simulation (97–101% of the dose in all simulations).

#### Sensitivity Analysis

Parameter sensitivity analysis was performed for the final IgG model (400 μl and 10.1 Torr) (Table I). Initially, diffusion coefficients were changed individually. The model was more sensitive to D_RPE-cho_ than to D_ret_ since RPE-choroid was the rate-limiting barrier with the lower diffusion coefficient for IgG. Unexpectedly, model was even more sensitive to the diffusion coefficient of IgG in vitreous (D_vit_; originally the same as D_wat_). For example, doubling D_vit_ enhanced the diffusion of IgG from the vitreous to the anterior eye and led to 13% increase in AH AUC, and 39% decrease in both vitreal and retinal AUC. With re-estimation of D_ret_ and D_RPE-cho_ the corresponding changes in D_vit_ caused lower changes in vitreal and retinal AUC than without re-estimation because of the large counteracting changes in estimated D_ret_ and D_RPE-cho_. However, at the same time, the changes in AH AUC were larger than without re-estimation leading to a marked change in the percentage of anterior elimination.

**Table I:** Sensitivity analysis of IgG model parameters

Changed parameter (original value)	Value (% of original)	Area under curve (AUC) (relative change from the final model as %)			Anterior^a^ elimination (% of dose)	Re-estimated parameters (relative change from the final model as %)	
		Vitreous	Retina	AH		D_ret_	D_RPE-cho_
Change in one parameter							
D_ret_ (36.8 × 10^−13^ m^2^s^−1^)	50 200	+0.8 −0.7	−1.4 +0.6	+1.0 −0.6	76.9 75.6	- -	- -
D_RPE-cho_ (4.11 × 10^−13^ m^2^s^−1^)	50 200	+10.2 −14.6	+13.1 −18.6	+11.4 −16.3	84.7 63.6	- -	- -
D_vit_ (6.73 × 10^−11^ m^2^s^−1^)	50 200	+52.8 −38.7	+50.1 −38.8	−22.9 +13.3	58.5 86.0	- -	- -
Change in one parameter with re-estimation of D_ret_ and D_RPE-cho_							
D_vit_ (6.73 × 10^−11^ m^2^s^−1^)	50 200	+3.3 −30.0	−5.3 −26.8	−50.7 +31.2	37.3 99.6	+1140 −85.4	+181 −99.9
K_ret/vit_ (0.5)	150 200	−2.0 −0.7	−4.8 +7.7	−2.3 −0.8	74.3 75.3	−94.6 −97.0	+165 +732
Weights (0.333/0.333/0.333 Vitreous/Retina/AH)	0.01/0.01/0.98^b^ 0.001/0.001/0.998	+4.0 +12.3	+0.3 +12.2	+4.6 +13.8	79.6 86.5	−68.6 −73.6	−13.3 −55.9

^a^Absolute value as % of dose (this was 76% of dose in the final model). The relative change was the same as for AUC in AH

^b^The weights in least squares method are given as absolute values

Abbreviations: AH is aqueous humor; D_ret_, D_RPE-cho_, and D_vit_ are diffusion coefficients of IgG in retina, combined retinal pigment epithelium-choroid, and vitreous, respectively. Originally, D_vit_ was the same as the diffusion coefficient in water (D_wat_). K_ret/vit_ is neural retina/vitreous partition coefficient at the boundary of these tissues

The increase in K_ret/vit_ from 0.5 to 1 (200% of the original) to abolish the sharp concentration drop at the retina-vitreous boundary caused only minor changes in AUC values, but caused 97% reduction in D_ret_ and 732% increase in D_RPE-cho_. This was related to the formation of concentration gradients in the neural retina and RPE-choroid, and these features are discussed in detail in Electronic Supplementary Material (Chapter 8).

With the final IgG model four of the five calculated AH concentrations were lower than the corresponding measured concentrations (Fig. 4c). When the weighting in the least squares method were changed aggressively to favor more accurate fit in AH (0.998 in AH versus 0.001 in both vitreous and retina), AH AUC increased by 14% with almost similar changes in vitreal and retinal AUC. These changes originated from 74% and 56% reductions in estimated D_ret_ and D_RPE-cho_, respectively.

### IVT Injection of Fab

**Fig. 7:**
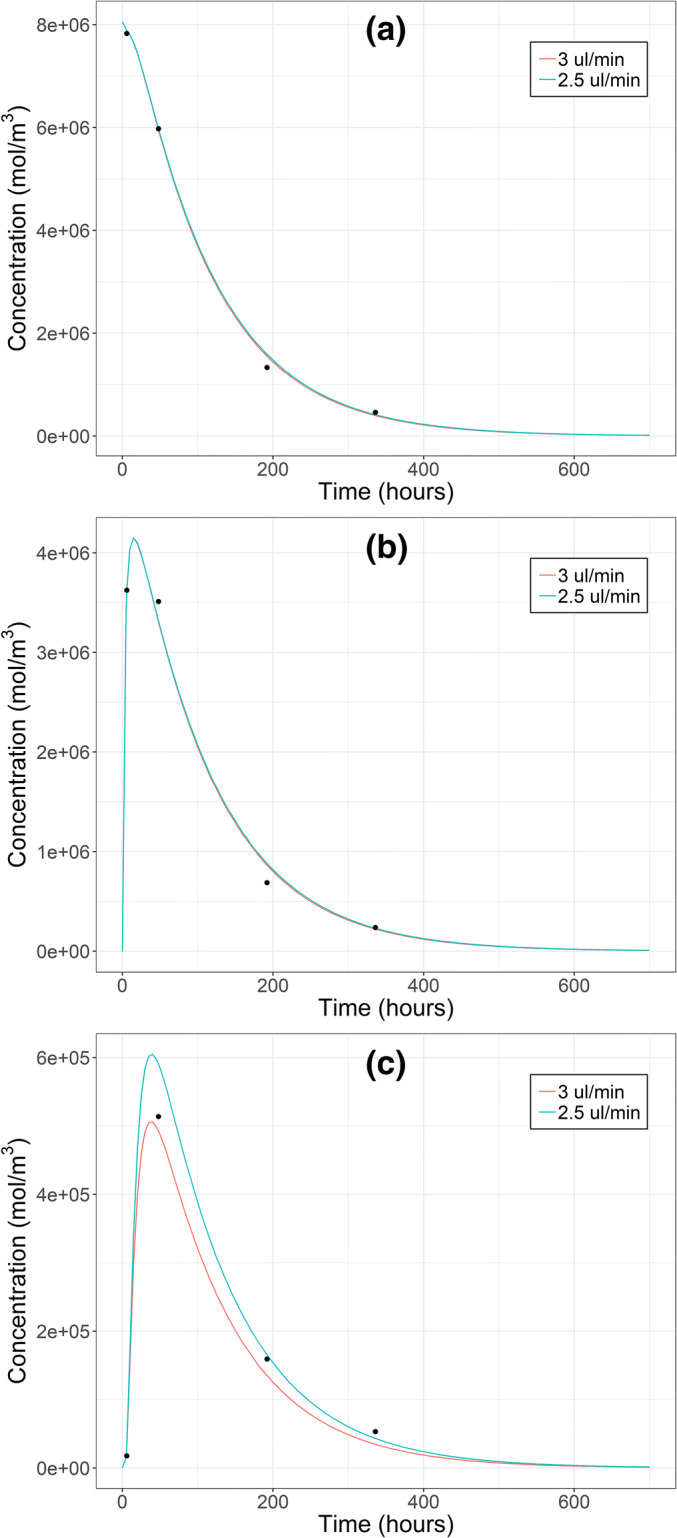
Calculated Fab concentrations after intravitreal injection in vitreous (**a**), retina (**b**) and aqueous humor (**c**) with different aqueous humor formation rates (lines) and measured concentrations (black dots, 26). Intraocular pressure was 10.1 Torr and initial distribution volume 400 μl. Note the different concentration scales in each panel

The final model for IgG (150 kDa) was adjusted to Fab (50 kDa) by using the appropriate values of D_wat_ (13.2 × 10^−7^ cm^2^s^−1^) and K_ret/vit_ (0.55) for Fab. This resulted in a good fit for Fab, except AH concentrations were slightly underestimated (Fig. 7). The reason for this underestimation was related to pharmacokinetic principles. In order to produce the experimentally determined AUC in AH (3.15 day nmol ml^−1^) with AH formation rate of 3 μl min^−1^ (4.32 ml day^−1^; representing also clearance from anterior chamber), the amount of Fab that needs be eliminated via anterior chamber is 13.6 nmol (the product of AUC and clearance). This is 111% of dose leading to a theoretical contradiction. However, when AH formation rate was reduced to 2.5 μl min^−1^, this contradiction was resolved leading to slightly higher AH concentrations (Fig. 7). With this final Fab model, the estimated D_ret_ and D_RPE-cho_ were 4.96 × 10^−9^ cm^2^ s^−1^ and 1.03 × 10^−9^ cm^2^ s^−1^, respectively, and the percentage of dose eliminated anteriorly was 96%.

## Discussion

The general aim of this study was to extend our earlier 3D ocular PK model (15) for parameter estimation, especially for estimating the diffusion coefficients of IgG and its antigen-binding fragment Fab in the neural retina (D_ret_) and combined retinal pigment epithelium-choroid (D_RPE-cho_) of the rabbit eye. The new features of handling neural retina and RPE-choroid as separate domains and adding neural retina/vitreous partition coefficient (K_ret/vit_) at the boundary of these tissues enabled the intended barrier analysis in the posterior eye segment.

With the formal least squares method, D_ret_ and D_RPE-cho_ were estimated for IgG at several K_ret/vit_ values using simultaneously experimental IgG concentrations in the rabbit vitreous, retina and AH. In the final IgG model, K_ret/vit_ was set to 0.5 based on the experimental ratio of mean retinal concentration to mean vitreal concentration (0.47) by Gadkar et al. (26), and estimated D_ret_ (36.8 × 10^−9^ cm^2^ s^−1^) was 9-fold compared with D_RPE-cho_ (4.11 × 10^−9^ cm^2^ s^−1^). This means that the RPE, the main barrier in the RPE-choroid layer, is a tighter barrier than retina with its ILM, as supported by the experimental data (3). When K_ret/vit_ was set to 1 to abolish the sharp concentration drop at the retina-vitreous boundary, the situation changed completely as estimated D_ret_ (1.1 × 10^−9^ cm^2^ s^−1^) was only 3% of D_RPE-cho_ (34.2 × 10^−9^ cm^2^ s^−1^), which was an unrealistic result (Table I and Electronic Supplementary Material).

In our opinion, K_ret/vit_ is an essential element in our model structure, and it can be adjusted based on the available experimental data. The biological explanation for the lower mean IgG concentration in the retina compared to vitreous is probably related to the barrier function of the ILM (29) and the fact that the neural retina consists mostly of different types of cells as opposed to the gel-like vitreous. For example, if the extracellular IgG concentration in the retina was the same as the total concentration in the vitreous and intracellular IgG concentration in the retina was significantly lower because of limited cellular uptake, the mean IgG concentration in retina would be markedly lower than in vitreous. Considering the large molecular size of IgG, this is a realistic scenario.

We studied the effect of the apparent initial distribution volume of IgG dose on the goodness of fit. The best fit was obtained with 400 μl even though the real injection volume was 50 μl. In our axisymmetric model, the spherical dosing volume with a uniform concentration was placed at the symmetry axis, and, thereby, cannot accurately model the true initial placement and mixing of the dose and the related variability in in vivo studies. In any case, our results showed that the initial distribution has to be taken into account and modeled. Earlier, the best fit for ranibizumab after 50 μl IVT injection was obtained by using 400 μl initial distribution volume that settled at the bottom of the eye due to the formulation’s higher specific gravity (21). The same study found that the long-term drug concentration profiles after the early time points seemed to depend only on the injected dose, and not on the initial distribution volume and its placement (21). We got a similar result with different initial volumes. We also found that there were up to 6-fold differences in the estimated D_ret_ and only minor differences in estimated D_RPE-cho_, when the initial volume was varied between 50 and 400 μl at IOP of 10.1 Torr.

Similar to our previous study (15), our model gave the best fit for macromolecules after IVT injection at 10.1 Torr where the AH flow toward posterior eye and through retina and RPE was minimal (0.001 μl/min of the total AH formation rate of 3 μl/min; Chapter 5 in Electronic Supplementary Material). However, our model cannot be used to make firm conclusions about the existence of posteriorly directed flow, since it is not meant for this purpose. The complexity in the determination of posteriorly directed flow was recently discussed in detail (30).

Sensitivity analysis revealed that the final IgG model was fairly sensitive to the changes in diffusion coefficient of IgG in the vitreous (D_vit_). After changes in D_vit_ re-estimated D_ret_ and D_RPE-cho_ differed markedly from those obtained with the final model (Table I). Therefore, D_vit_ for each drug has to be chosen with care for mathematical modeling as discussed recently (30). Both the size (Stokes-Einstein equation) and the net charge of the macromolecule affect its diffusion rate in the vitreous. Several studies have established that the vitreal diffusion of positively charged macromolecules and nanoparticles is restricted because of electrostatic interaction with the negatively charged hyaluronic acid molecules abundantly found in the vitreous (31–34). Moreover, the average mesh size of bovine vitreous has been estimated at about 550 nm; above this size particles are immobilised because of steric hindrance from the vitreous meshwork (32).

Hutton-Smith et al. (16) used the same IgG and Fab data earlier to estimate permeability in ILM and RPE with a semi-mechanistic 3-compartment model. They estimated that the percentage of the anterior route was 82% for IgG and 87% for Fab, respectively, which are close to our estimates (76% and 96%). Their IgG model is compared with our model in Electronic Supplementary Material (Chapter 8) where diffusion coefficients are converted to permeability values to make the comparison transparent. While the calculated concentration profiles in retina, RPE and choroid were quite different in these models, the total permeability across these layers was practically the same, and, therefore, both models fitted the experimental concentrations well. However, in our opinion, the permeability estimates obtained with our model (retinal permeability including ILM was higher than RPE-choroid permeability) were more realistic than those obtained by Hutton-Smith et al. (16) (equal permeability for ILM and RPE with well-stirred retina in between).

In Fab modeling, a pharmacokinetic contradiction appeared with AH formation rate of 3 μl/min. The experimentally determined AUC of Fab in AH could not be obtained with the model even if the whole dose was eliminated via the anterior chamber. The pharmacokinetic calculations related to this issue are described shortly in the results, and in more detail in our earlier study and its supplementary (15). The contradiction was resolved technically by reducing AH formation rate to 2.5 μl/min. In reality, the best option would be to determine AH formation rate and its diurnal variability in the animals that will used in PK study. Equally important for correct mass balance is accurate IVT dosing and prevention of its leakage after injection, and accurate drug concentration measurements from the ocular tissues. The geometric dimensions of the virtual eye in the model should also be as close as possible to the real animal or human eye in the PK study.

Even though we reduced AH formation rate in Fab model to 2.5 μl/min to abolish the need to eliminate the whole dose via the anterior chamber, estimated D_ret_ (4.96 × 10^−9^ cm^2^ s^−1^) and D_RPE-cho_ (1.03 × 10^−9^ cm^2^ s^−1^) for Fab were 7 and 4 times lower than those for IgG, respectively. Based on molar masses of Fab (50 kDa) and IgG (150 kDa) alone, the opposite results would be expected as a smaller molecule typically penetrates the posterior eye membranes faster than a bigger molecule (3,35). It is possible that the binding, uptake or permeation mechanism of Fab differs from IgG. The unexpected result may also arise from the inaccuracy of the actual delivered dose or the measured tissue concentrations since an exact mass balance is the basis for meaningful results. Hutton-Smith et al. (16) used AH formation rate of 3 μl/min for both Fab and IgG, and their best estimates of ILM and RPE permeability for Fab were slightly higher than for IgG while 95% confidence intervals were largely overlapping. However, it seems that their model underestimated Fab concentrations in AH (see Fig. 2 in 16).

For parameter estimation our computational fluid dynamics (CFD) model is inherently more complicated than the compartmental model by Hutton-Smith et al. (16). On the other hand, our model offers several advantages in terms of physiologically-based modeling. For example, Hutton-Smith et al. (16) needed an additional virtual delay chamber to move the peak concentration in AH from time zero to the correct time. They also had to estimate an elimination rate constant from vitreous to aqueous chamber, while in our model this mass transfer was governed by the underlying convection and diffusion. Ocular CFD models based on animal data can also be scaled to human with physiological principles (20,21).

Regarding the limitations in our study, the posterior eye in our model consisted of retina and combined RPE-choroid to keep the model structure simple for parameter estimation, and, thereby, partly lacked anatomical and physiological relevance. A more detailed model of the posterior eye has been built (21), but its use for parameter estimation would probably require fixing of several parameters based on prior knowledge. In the actual regression, we used mean concentration data instead of all individual data points and omitted day 21 vitreal and retinal concentrations for Fab due to missing AH concentrations and related technical problems (Table S1 in Electronic Supplementary Material). Additionally, COMSOL software did not give standard errors for parameters estimates which are normally obtained with regression software and used for the evaluation of goodness of fit. A general limitation was the lack of physiological data on AH flow. AH formation rate in rabbits was not determined by Gadkar et al. (26), and we used a literature value for IgG (3 μl/min) and an adjusted value for Fab (2.5 μl/min), respectively. As discussed above there is no consensus on the posteriorly directed AH flow, and, therefore, we performed simulations with multiple IOP values to obtain different AH flow patterns. Experimental data on these physiological phenomena would provide a more solid basis for the modeling.

## Conclusion

Our previously published 3D ocular PK model for IVT injection in the rabbit eye was extended to estimate the diffusion coefficients of IgG antibody and its antigen-binding fragment Fab in neural retina and the combined retinal pigment epithelium-choroid. This study showed that 3D ocular PK models can be used for challenging parameter estimation tasks using simultaneously macromolecule concentrations in several ocular tissues. This method is a valuable tool for data analysis and interpretation. The model can be used also for other antibodies and scaled to human eye.

### Acknowledgments and disclosures

Financial support was obtained from the Graduate school of pharmacy of the University of Eastern Finland, Finnish Pharmaceutical Society, Sokeain Ystävät – De Blindas Vänner sr, Santen, Academy of Finland (project 311122), and the Lundbeck Foundation (grant R181-2014- 3577). TK is a former employee of COMSOL OY, Helsinki, Finland. The other authors declare that they have no conflict of interests.

## Electronic supplementary material

ESM 1 (PDF 1234 kb)

## Abbreviations

2D/3D: Two/three-dimensional
AH: Aqueous humor
AMD: Age-related macular degeneration
AUC: Area under the curve
C_calc,t_: Calculated mean concentration of macromolecule in corresponding domain at time t
CFD: Computational fluid dynamics
C_obs,t_: Observed mean concentration of macromolecule in corresponding domain at time t
D_ret_: Diffusion coefficient of macromolecule in neural retina (cm^2^ s^−1^)
D_RPE-cho_: Diffusion coefficient of macromolecule in retinal pigment epithelium-choroid (cm^2^ s^−1^)
D_vit_: Diffusion coefficient of macromolecule in vitreous (cm^2^ s^−1^)
D_wat_: Diffusion coefficient of macromolecule in water (cm^2^ s^−1^)
Fab: IgG’s antigen-binding fragment
FEM: Finite element modeling
IgG: Immunoglobulin G
ILM: Inner limiting membrane
IOP: Intraocular pressure (Torr)
IVT: Intravitreal
K_ret/vit_: Neural retina/vitreous partition coefficient
OFV: Objective function value
P_app_: Apparent permeability coefficient of macromolecule in membrane (cm s^−1^)
P_ILM_: Apparent permeability coefficient of macromolecule in inner limiting membrane (cm s^−1^)
PK: Pharmacokinetics
P_RPE_: Apparent permeability coefficient of macromolecule in retinal pigment epithelium (cm s^−1^)
r_H_: Hydrodynamic radius (nm)
RPE: Retinal pigment epithelium
SSR: Sum of squared residuals
t_1/2_: Elimination half-life (h)
TM: Trabecular meshwork
V_init_: Initial distribution volume of the dose (μl)
W: Weight of a domain in the least-squares regression
