# Supplementary material for: Extended Pharmacokinetic Model of the Intravitreal Injections of Macromolecules in Rabbits. Part 2: Parameter Estimation Based on Concentration Dynamics in the Vitreous, Retina, and Aqueous Humor
Source: Pharm Res. 2020 Oct 22;37(11):226. doi: 10.1007/s11095-020-02946-1 (PMC7581578; doi:10.1007/s11095-020-02946-1)
Supplement: Supplementary file 1 — (PDF 1234 kb) [file 11095_2020_2946_MOESM1_ESM.pdf]

ELECTRONIC SUPPLEMENTARY MATERIAL

Extended Pharmacokinetic Model of the Intravitreal Injections of Macromolecules in Rabbits. Part 2: Parameter Estimation Based on Concentration Dynamics in the Vitreous, Retina, and Aqueous Humor

Lamminsalo Marko[a], Karvinen Timo[b], Subrizi Astrid[a], Urtti Arto[a,c,d], Ranta Veli-Pekka[a]

[a] School of Pharmacy, Faculty of Health Sciences, University of Eastern Finland, Kuopio, Finland

[b] Granlund Consulting Oy, Helsinki, Finland

[c] Division of Pharmaceutical Biosciences, Faculty of Pharmacy, University of Helsinki, Helsinki, Finland

[d] Laboratory of Biohybrid Technologies, Institute of Chemistry, St. Petersburg State University, St. Petersburg, Russian Federation

Contents

1. Experimental data for parameter estimation
2. Equations for fluid flow, mass transfer and heat transfer
3. Boundary conditions
4. Initial distribution of dose
5. Aqueous humor outflow at different intraocular pressures
6. Péclet numbers
7. Estimated diffusion coefficients and percentage of anterior elimination of IgG in all combinations of intraocular pressure and initial distribution volume
8. Effect of neural retina/vitreous partition coefficient on IgG concentration gradients and diffusion coefficients, and comparison with the model by Hutton-Smith et al. (2017)
9. Effect of intraocular pressure on estimated parameters and elimination pathways of IgG

References

1. Experimental data for parameter estimation

Table S1. PK datasets for IgG antibody and its antigen-binding fragment Fab.

Dataset (initial dose)	Time (d)	Concentration (pM)			Retina/Vitreous ratio*
		Retina	Vitreous	Aqueous	
IgG (0.549 mg)	0.25	1120012	2377123	31791.67	0.471
	3	796927.4	1698175	64868.83	0.469
	9	385239.8	790833.2	51239.17	0.487
	15	140409.3	336675.2	20432.33	0.417
	29	29675.71	59392.5	2664	0.500
Fab (0.615 mg)	0.25	3624653	7829061	17829.77	0.463
	2	3510730	5977675	513850.4	0.587
	8	688210	1332710	159342.2	0.516
	14	237846.7	460254.5	53152.1	0.517
	21	25580	85261.3	-	0.300

* Calculated based on presented data

Original data of Gadkar et al. (2015), explicitly presented in supplementary information of Hutton-Smith et al. (2017). Concentrations at respective time points have been averaged. Fab concentration data from day 21 was omitted in parameter estimation since the handling of missing aqueous data was not possible with COMSOL software.

2. Equations for fluid flow, mass transfer and heat transfer

From Lamminsalo et al. (2018), except the slightly modified Table S2

Based on the Navier-Stokes equations, the equations used in the model to describe incompressible flow of single-phase fluid (anterior and posterior chambers) are

$$\rho(\boldsymbol{u} \cdot \nabla)\mathbf{u} = \nabla \cdot [-\mathrm{p}\mathbf{I} + \mu(\nabla\mathbf{u} + (\nabla\mathbf{u})^{\mathrm{T}})] + \boldsymbol{F} + (\rho - \rho_{ref})\boldsymbol{g}$$

$$\rho\nabla \cdot \boldsymbol{u} = 0$$

where

- ρ is the density (SI unit: kg/m^3)
- **u** is the velocity vector (SI unit: m/s)
- p is pressure (SI unit: Pa)
- **I** is the identity matrix
- μ is the dynamic viscosity of the fluid (SI unit: kg/(m·s))
- **F** is the volume force vector (SI unit: N/m^3)
- **g** is the gravity vector from anterior to posterior eye along symmetry axis (SI unit: m/s^2)
- C_p is the specific heat capacity at constant pressure (SI unit: J/(kg·K))
- T is the absolute temperature (SI unit: K)

The former represents the momentum and the latter the continuity equation. In COMSOL Multiphysics, this form of Boussinesq approximation was obtained by defining the fluid as

incompressible with the "include gravity" and "use reduced pressure" options selected, and coupling the heat transfer and flow interfaces with Nonisothermal Flow multiphysics feature.

For porous media (vitreous, neural retina, RPE-choroid, sclera, trabecular meshwork, cornea), the Brinkman equations were used:

$$\frac{1}{\varepsilon_p}\rho(\boldsymbol{u}\cdot\nabla)\mathbf{u}\frac{1}{\varepsilon_{\mathrm{p}}} = \nabla\cdot[-\mathrm{p}\mathbf{I} + \mu\frac{1}{\varepsilon_{\mathrm{p}}}\left(\nabla\mathbf{u} + (\nabla\mathbf{u})^{\mathrm{T}}\right)] - \left(\mu\boldsymbol{\kappa}^{-1} + \frac{Q_{br}}{\varepsilon_p^2}\right)\boldsymbol{u} + \boldsymbol{F}$$

$$\rho\nabla\cdot\boldsymbol{u} = Q_{br}$$

where
- ε_p is the porosity
- $\boldsymbol{\kappa}$ is the permeability tensor of the porous medium (SI unit: m^2)
- Q_{br} is mass source of mass sink (SI unit: $kg/(m^3\cdot s)$)

Heat transfer in the model (in the whole eye) is described with the equation

$$\rho\, C_p\boldsymbol{u}\cdot\nabla T + \nabla\cdot\boldsymbol{q} = Q$$

$$\boldsymbol{q} = -k\nabla T$$

where
- ρ is the density (SI unit: kg/m^3)
- C_p is the specific heat capacity at constant pressure (SI unit: J/(kg·K))
- T is the absolute temperature (SI unit: K)
- **u** is the velocity vector (SI unit: m/s)
- **q** is the heat flux by conduction (SI unit: W/m^2)
- k is the thermal conductivity (SI unit: W/(m·K))
- α_p is the coefficient of thermal expansion (SI unit: 1/K):

$$\alpha_p = -\frac{1}{\rho}\left(\frac{\partial\rho}{\partial T}\right)_\rho$$

- p is the pressure (SI unit: Pa)
- Q contains additional heat sources (SI unit: W/m^3)

Heat transfer and Laminar flow were coupled in Non-isothermal flow physics in which the effect of temperature-dependent material properties, such as dynamic viscosity, density and thermal expansion, in the flow velocity was regarded.

Mass transport with diffusion and convection (vitreous, neural retina, RPE-choroid, anterior and posterior chamber and trabecular meshwork) was described with equation

$$\frac{\partial \mathrm{c_i}}{\partial \mathrm{t}} + \nabla\cdot(-D_i\nabla c_i + \boldsymbol{u}c_i) = 0$$

$$\boldsymbol{N_i} = -D_i\nabla c_i + \boldsymbol{u}c_i$$

where
- c_i is the concentration of the species (SI unit: mol/m^3)
- D_i denotes the diffusion coefficient (SI unit: m^2/s)
- **u** is the velocity vector (SI unit: m/s)
- **N** is the flux vector (SI unit: $mol/(m^2\cdot s)$)

The bolus injections are defined as initial concentrations in the corresponding domain.

Table S2. Summary of mass transfer coefficients in IgG and Fab models.

Tissue	Model parameter shown in Table II
Vitreous	D_{vit}=D_{wat}
Aqueous humor	D_{wat}
Trabecular meshwork	D_{wat}
Neural retina	D_{ret}
RPE-Choroid	$D_{RPE\text{-}cho}$
Lens, ciliary body, iris and corna	Impermeable to macromolecules

D = diffusion coefficient; wat = water

3. Boundary conditions

From Lamminsalo et al. (2018)

No slip, the default boundary condition was set to tissues defined as solid (lens, iris, ciliary body). In no slip boundaries the fluid velocity relative to the boundary is zero, i.e. $\boldsymbol{u} = 0$. The inlet in the posterior chamber was described with velocity boundary condition

$$\boldsymbol{u} = -U_0\boldsymbol{n}$$

where U_0 is the normal inflow velocity (SI unit: m/s) and **n** is the normal vector of the inlet wall pointing out of the domain. For the inlet, U_0 is set to 2.219×10^{-6} m/s. The outlets at Schlemm's canal, sclera and cornea were described with pressure boundary condition

$$[-p\boldsymbol{I} + \mu(\nabla\boldsymbol{u} + (\nabla\boldsymbol{u})^T]\boldsymbol{n} = -\hat{p}_0\boldsymbol{n}$$

$$\hat{p}_0 \leq p_0$$

where p_0 is the relative pressure at the boundary. For Sclemm's canal and sclera, p_0 is set to 1333.22 Pa (10 Torr) and for cornea to 0 Pa. Backflow from outlets is suppressed.

For heat transfer, the temperature was set using the boundary condition

$$T = T_0$$

where T_0 is the set temperature (SI unit: K). For sclera and fluid inlet the temperature is set to 37 ^{o}C and for cornea 34 ^{o}C. Outflow of heat is allowed with convection through the Schlemm's canal described by

$$-\boldsymbol{n} \cdot \boldsymbol{q} = 0.$$

In mass transport, the concentration boundary condition used in the model where only diffusion through neural retina and RPE-choroid was allowed was set as

$$c = c_0$$

With only diffusion, the boundary condition for flux is given by equation

$$N = k_c(c_b - c)$$

where N is the flux expression (SI unit: mol/(m^2·s)), k_c mass transfer coeffiecient (SI unit: m/s) and c_b the concentration in the surroundings of the modelled system ('bulk concentration').

Assuming convection is the governing transport mechanism, the outflow can be described ignoring diffusion such that

$$\boldsymbol{n} \cdot (-D\nabla c) = 0.$$

4. Initial distribution of dose

Table S3. Key radii and coordinates (cm) for the intravitreal dose spheres in rabbit eye.

Volume of intravitreal dose sphere (µl)	R1	R2	X-cent	Z-cent
50	0.229	-	0	0.339
250	0.455	0.288	0	0.377
400	0.576	0.288	0	0.377

Volume is obtained after rotating the sphere around the symmetry axis.
Coordinates given in similar form as in Lamminsalo et al. (2018).

Table S4. Initial concentrations for 2D-axisymmetric geometry to get matching dose to *in vivo* data.

Initial distribution volume (µl)	Initial conc in dose sphere (mol m^{-3})	
	IgG	Fab
50	7.32×10^7	2.46×10^8
250	1.46×10^7	4.92×10^7
400	9.15×10^6	3.08×10^7
1516	2.41×10^6	8.11×10^6

5. Aqueous humor outflow at different intraocular pressures

In this mathematical model, intraocular pressure was used to change the aqueous humor outflow pattern to theoretically analyze different scenarios, and these settings are not intended to describe the effect of IOP on real physiological phenomena.

Table S5. Aqueous humor outflow through trabecular meshwork (TM), retina and cornea at different intraocular pressures (IOP). Aqueous humor formation rate was 3 µl min^{-1}.

Intraocular pressure (Torr)[a]	TM hydraulic permeability ($\times 10^{-16}$ m^2)	Outflow (µl min^{-1})			Fluid velocity at retinal surface[b] ($\times 10^{-7}$ cm s^{-1})
		TM	Retina	Cornea	
10.1	202.5	2.917	0.001	0.060	0.034
12.5	7.797	2.878	0.025	0.075	0.84
15	3.840	2.837	0.051	0.091	1.7
17.5	2.522	2.796	0.076	0.107	2.6
20	1.863	2.755	0.102	0.123	3.4

[a] The unit Torr is equivalent to the unit

[b] Mean value obtained by dividing the outflow through retina by the retinal surface area (4.96 cm^2).

From Lamminsalo et al. (2018)

6. Péclet numbers

The ratio of convective flux and diffusive flux in mass transfer can be measured with a dimensioless number, the Péclet number (Pe), with equation

$$Pe = \frac{convection}{diffusion} = \frac{u \times L}{D}$$

where

• u is the local convective fluid flow velocity (SI unit: m/s)

• L is a characteristic length scale (SI unit: m)

• D denotes the diffusion coefficient (SI unit: m^2/s)

When Pe > 1 convection dominates, when Pe < 1 diffusion dominates and when Pe ≈ 1 their effect is similar. The calculated Péclet numbers for IgG (Figure S1, Table S6) show that the transport in anterior and posterior chambers is governed by convection while diffusion dominates in the vitreous. At intraocular pressure values between 10.1 and 20 Torr, the flow velocity values u are practically similar in the anterior and posterior chambers, respectively, while the flow velocity in the vitreous has 5-fold difference (Table S6). The Péclet numbers in the vitreous are still markedly below 1 in both instances (Table S6). The case is similar with Fab (D = 13.2×10^{-7} cm^2s^{-1}).

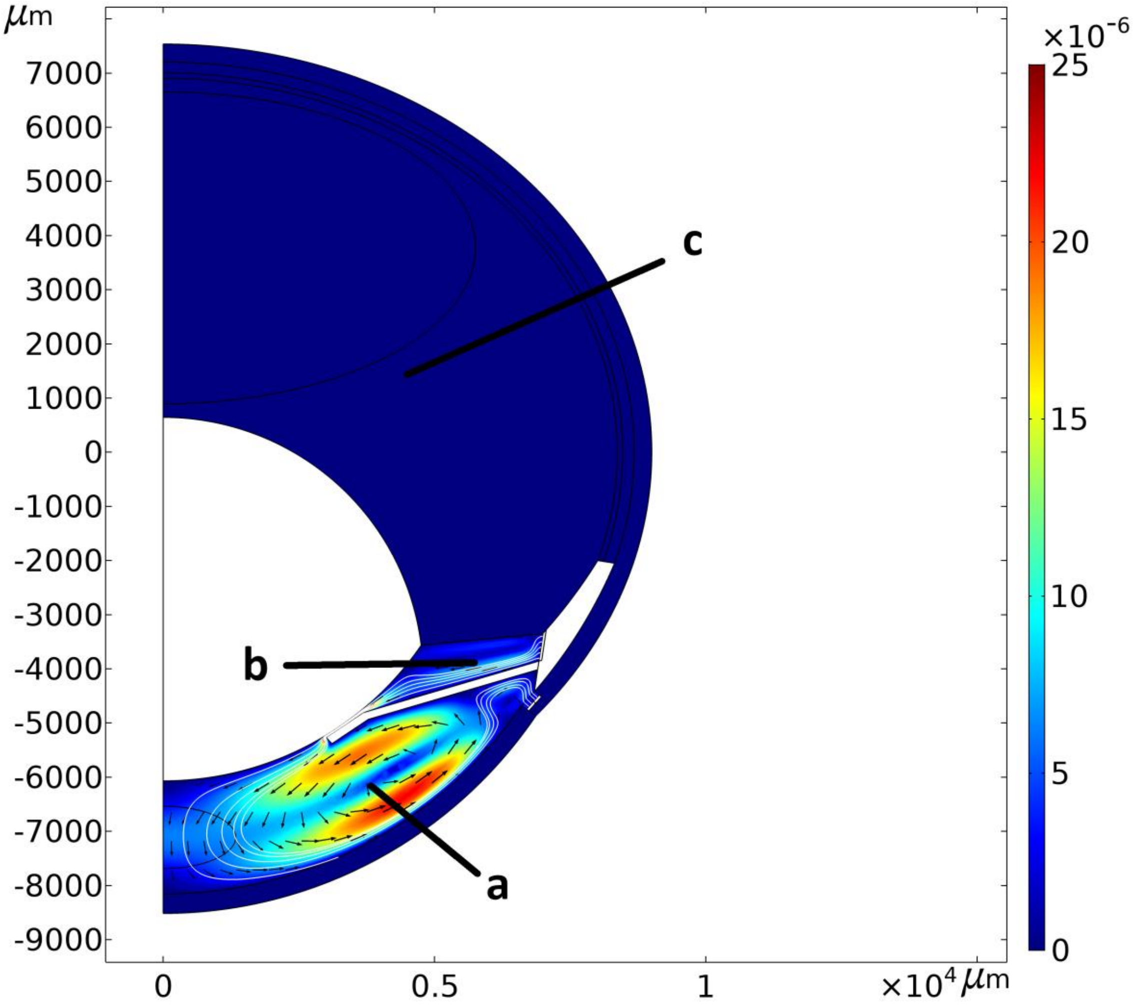

Figure S1 Aqueous humor velocity (m s^{-1}) and flow pattern at 10.1 Torr with denoted positions for calculating the Péclet numbers for IgG in (**a**) anterior chamber, (**b**) posterior chamber and (**c**) vitreous. Péclet numbers were calculated using average flow velocities (Table S6). Note that the maximum flow velocities are substantially larger, e.g. in the anterior chamber (**a**) the maximum flow velocity in the whirl is 2.2×10^{-3} cm s^{-1} which yields Péclet number of 1210.

Table S6. Péclet numbers for IgG in the rabbit eye at intraocular pressures 10.1 and 20 Torr.

Position	u (cm/s)	L (cm)	D (10^{-7} cm^{2}s^{-1})	Pe	Transport driven by
Anterior chamber					
- 10.1 and 20 Torr	9.8×10^{-4}	0.37[a]	6.73	540	convection
Posterior chamber					
- 10.1 and 20 Torr	4.4×10^{-4}	0.13[b]	6.73	85	convection
Vitreous					
- 10.1 Torr	1.4×10^{-7}	0.626[c]	6.73	0.13	diffusion
- 20 Torr	6.9×10^{-7}	0.626[c]	6.73	0.64	diffusion

Flow velocity in the corresponding domain (Fig. S1) is expressed as avg u (average).

[a] distance from the tip of iris to trabecular meshwork

[b] half of the distance between the lens equator and the aqueous humor inlet

[c] distance between the posterior lens and neural retina on the symmetry axis

7. Estimated diffusion coefficients and percentage of anterior elimination of IgG in all combinations of intraocular pressure and initial distribution volume

Table S7. Estimated parameters and percentage of anterior elimination of IgG.

IOP [Torr]	Initial volume [µl]	D_{ret} [10^{-9} cm^2 s^{-1}]	$D_{RPE-cho}$ [10^{-9} cm^2 s^{-1}]	Anterior elimination (% of dose)
**10.1**	50	105	5.02	73.6
	250	223	4.33	74.5
	400	36.8	4.11	76.0
	1516	8.50	0.001	98.0
**12.5**	50	120	6.39	66.0
	250	366	5.57	67.1
	400	31.7	5.52	68.6
	1516	9.72	0.811	92.6
**15**	50	167	7.57	58.9
	250	228	6.77	59.9
	400	28.2	6.90	61.6
	1516	10.5	2.45	83.7
**17.5**	50	494	8.56	52.3
	250	362	7.82	53.2
	400	25.1	8.26	55.0
	1516	11.5	3.93	75.8
**20**	50	423	9.54	46.1
	250	363	8.75	47.0
	400	23.5	9.55	48.9
	1516	12.8	5.61	67.7

8. Effect of neural retina/vitreous partition coefficient on IgG concentration gradients and diffusion coefficients, and comparison with the model by Hutton-Smith et al. (2017)

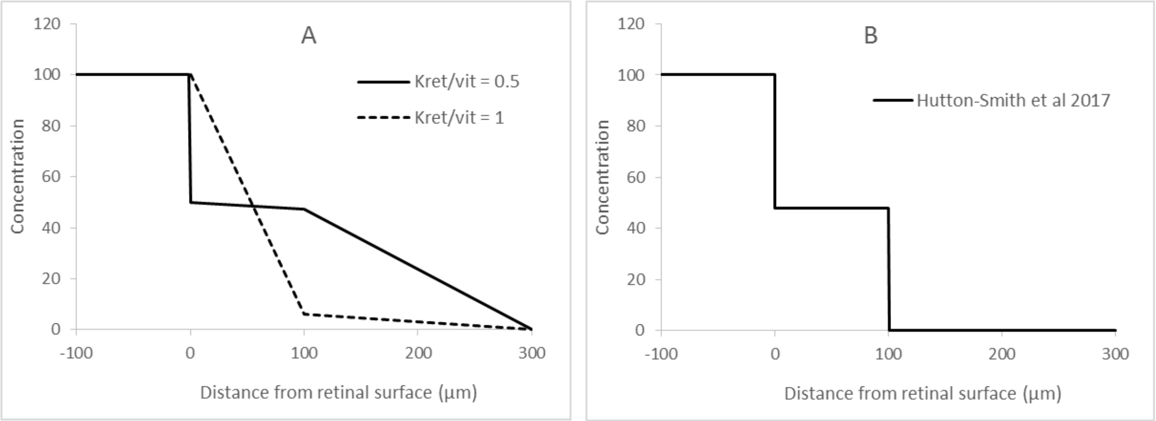

Figure S2 Illustration of concentration gradients of IgG at steady-state in the current study with neural retina/vitreous partition coefficient ($K_{ret/vit}$) of 0.5 and 1 (A) and in Hutton-Smith et al. (2017) (B). Distances in the current study: -100 µm = posterior vitreous close to retinal surface; 0 µm = retinal surface; 100 µm = surface of retinal pigment epithelium (RPE); 300 µm = surface of sclera. Combined RPE-choroid extends from 100 µm to 300 µm, and the sink is located at 300 µm. In Hutton-Smith et al. (2017), inner limiting membrane is at 0 µm (zero thickness), retina extends from 0 to 100 µm, and RPE is at 100 µm (zero thickness). The concentration of IgG in vitreous is kept constant at 100. The decline in IgG concentration in each membrane is directly proportional to its fractional resistance (Table S8). However, with $K_{ret/vit}$ of 0.5, the fractional resistances describe the concentration profile between the concentration of 50 at the retinal surface and the zero concentration at the sink, and in other cases across the total concentration difference (100), respectively.

Table S8. Parameter estimates for IgG in the current study and in Hutton-Smith et al. (2017)[a]

Model	D_{ret} (10^{-9} cm^2 s^{-1})	$D_{RPE-cho}$ (10^{-9} cm^2 s^{-1})	P_{ret} / P_{ILM} (10^{-7} cm s^{-1})	$P_{RPE-cho}$ / P_{RPE} (10^{-7} cm s^{-1})	P_{tot} (10^{-7} cm s^{-1})	Fractional resistance in retina / ILM (%)	Fractional resistance in RPE-cho / RPE (%)
$K_{ret/vit}$ = 0.5	36.8	4.11	36.8	2.06	1.95	5.3	94.7
$K_{ret/vit}$ = 1	1.10	34.2	1.10	17.1	1.04	93.9	6.1
Hutton-Smith	-	-	1.70	1.84	0.88	52.0	48.0

[a] Current study estimated parameters for retina (ret) and combined retinal pigment epithelium-choroid (RPE-cho), whereas Hutton-Smith et al. (2017) estimated parameters for inner limiting membrane (ILM) and RPE.
Abbreviation and calculations:
D, estimated diffusion coefficient in the current study
P, apparent permeability: in the current study, D was converted to P by dividing by the membrane thickness (100 µm for retina and 200 µm for RPE-choroid); estimated P from Hutton-Smith et al. (2017)
P_{tot}, total permeability across the two membrane barriers: $1/P_{tot} = 1/P_{ret} + 1/P_{RPE-cho}$
In the previous equation, each 1/P term represents diffusional resistance, and the equation was used to calculate the fractional resistance from the total resistance. With $K_{ret/vit}$ of 0.5, the fractional resistances describe the concentration profile between the concentration of 50 at the retinal surface and the zero concentration at the sink (Fig. S2), and in other cases across the total concentration difference (100), respectively.

In the current study, the model with $K_{ret/vit}$ of 0.5 was the final model for IgG, and other models are compared with it.

When $K_{ret/vit}$ was set to 1, the concentration of IgG at the retinal surface was equal to that at the posterior vitreous. In order to get the experimentally measured ratio of mean retinal concentration to mean vitreal concentration of approximately 0.5, the concentration at retina/RPE border needs to be very low. The result in the parameter estimation was that 94 % of the diffusional resistance was placed to retina, and, hence, diffusion coefficient and permeability of IgG in retina was much lower than in RPE-choroid. This contradicts with the literature where RPE is generally considered to be a tighter barrier for macromolecules than neural retina (del Amo et al. 2017). The total permeability with $K_{ret/vit}$ of 1 was about 50 % of that with $K_{ret/vit}$ of 0.5. This is because the total concentration difference described with the total permeability was double (100 versus 50 in Fig. S2). A double concentration difference combined with a halved total permeability in the model with $K_{ret/vit}$ of 1 produced the same flux though the barriers and similar calculated concentrations to our final IgG model with $K_{ret/vit}$ of 0.5 (see the similar AUC values in Table 1 of the manuscript).

Hutton-Smith et al. (2017) used the traditional well-stirred compartments and sharp concentration drops in ILM and RPE in their model. Because the experimentally determined retina/vitreous concentration ratio was approximately 0.5 and the calculated concentration profile in retina was flat (well-stirred), the concentration drops in ILM and RPE had to be practically equal, and, therefore, estimated permeability values in ILM and RPE were almost equal. The total permeability was close to that obtained in the current study with $K_{ret/vit}$ of 1, whereas it was approximately 50 % of that with $K_{ret/vit}$ of 0.5 for the reasons described above.

9. Effect of intraocular pressure on estimated parameters and elimination pathways of IgG

Table S9. Parameter estimates for IgG at different intraocular pressures (IOP)

IOP (Torr)	D_{ret} (10^{-9} cm^2 s^{-1})	$D_{RPE\text{-}cho}$ (10^{-9} cm^2 s^{-1})	P_{ret} (10^{-7} cm s^{-1})	$P_{RPE\text{-}cho}$ (10^{-7} cm s^{-1})	P_{tot} (10^{-7} cm s^{-1})	Fractional resistance in retina (%)	Fractional resistance in RPE-cho (%)
10.1	36.8	4.11	36.8	2.06	1.95	5.3	94.7
12.5	31.7	5.52	31.7	2.76	2.54	8.0	92.0
15	28.2	6.90	28.2	3.45	3.07	10.9	89.1
17.5	25.1	8.26	25.1	4.13	3.55	14.1	85.9
20	23.5	9.55	23.5	4.78	3.97	16.9	83.1

D, estimated diffusion coefficient

P, apparent permeability that was obtained by dividing D by the membrane thickness (100 µm for retina and 200 µm for RPE-choroid)

P_{tot}, total permeability across the two membrane barriers: $1/P_{tot} = 1/P_{ret} + 1/P_{RPE\text{-}cho}$

In the previous equation, each 1/P term represents diffusional resistance, and the equation was used to calculate the fractional resistance from the total resistance.

The model with the initial distribution volume of 400 microliters and neural retina/vitreous partition coefficient of 0.5 was used in parameter estimation.

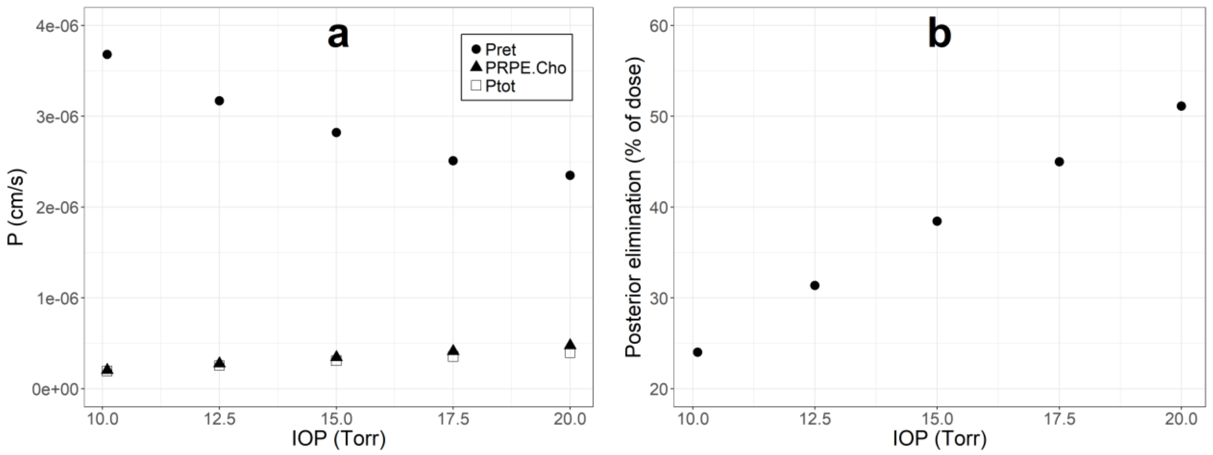

Figure S3 Estimated permeability of IgG in neural retina (circle) and RPE-choroid (triangle) and total permeability across both layers (open square) as a function of intraocular pressure (IOP) (a) and the corresponding percentage of dose eliminated posteriorly (b). The model with the initial distribution volume of 400 microliters and neural retina/vitreous partition coefficient of 0.5 was used in parameter estimation.

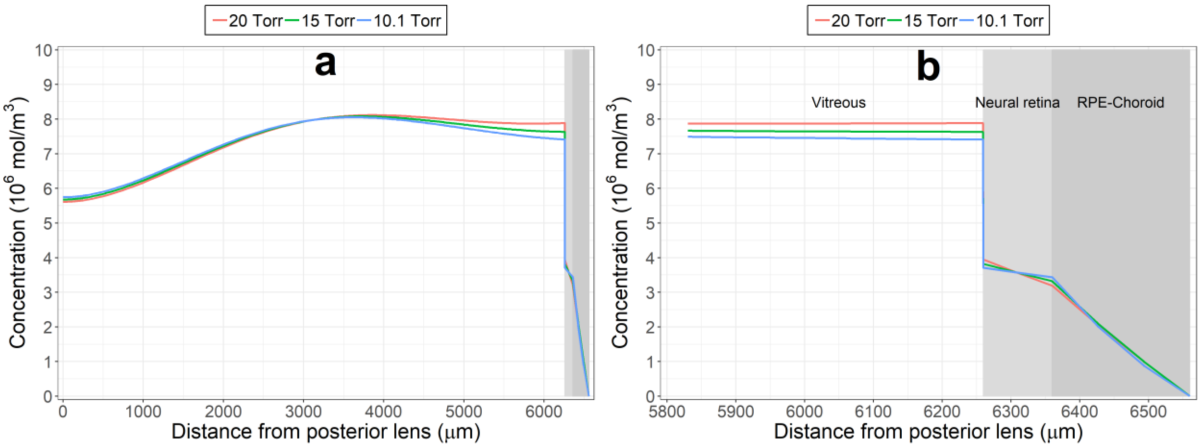

Figure S4 Calculated IgG concentrations at 7 h after intravitreal injection at different intraocular pressures. The concentrations are shown on the symmetry axis from the posterior lens (0 μm) to the sink at the anterior sclera (6560 μm) as whole (a) and enlarged for the posterior eye (b). The effect of neural retina/vitreous partition coefficient (0.5) can be observed at vitreous-retina boundary at distance 6260 μm.

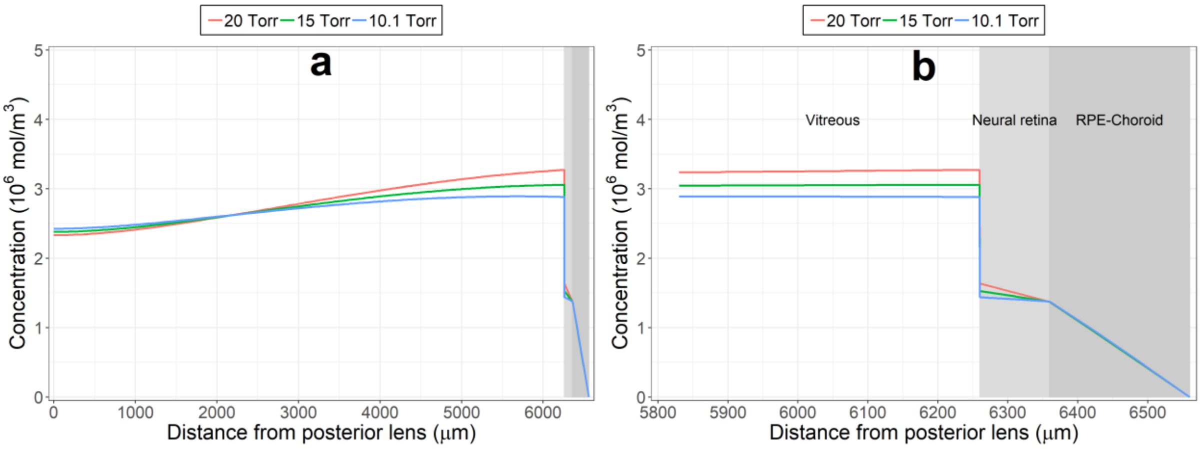

Figure S5 Calculated IgG concentrations at 70 h after intravitreal injection at different intraocular pressures. The concentrations are shown on the symmetry axis from the posterior lens (0 μm) to the sink at the anterior sclera (6560 μm) as whole (a) and enlarged for the posterior eye (b). The effect of neural retina/vitreous partition coefficient (0.5) can be observed at vitreous-retina boundary at distance 6260 μm.

In this mathematical model, an increase in IOP directed more aqueous humor through retina (Table S5). This convective transport of IgG toward retina hindered elimination via the anterior route. However, the transport of IgG through retina and RPE-choroid was purely diffusive since convective term in these layers were set to zero based on our earlier study (Lamminsalo et al. 2018). In the parameter estimation, a good match between measured and calculated concentrations in vitreous and retina was obtained by increasing the diffusion coefficient in RPE-choroid with IOP since this was the rate-limiting barrier in the posterior eye (Table S9 and Figure S3a). This also led to an increase in total permeability. However, the underestimation of aqueous humor concentrations got worse with increasing IOP (Figure 4 in the manuscript), and the percentage of dose eliminated via the posterior route increased markedly (Figure S3b). On the other hand, diffusion coefficient and permeability in retina decreased with increasing IOP thereby increasing the fractional resistance of retina (Table S9 and Figure S3a). This was necessary to create a steeper concentration decline in retina in order to prevent a significant increase in the calculated mean retinal concentration (Figures S4 and S5).

References

(1) Gadkar K, Pastuskovas CV, Le Couter JE, Elliott JM, Zhang J, Lee CV, Sanowar S, Fuh G, Kim HS, Lombana TN, Spiess C, Nakamura M, Hass P, Shatz W, Meng YG, Scheer JM. Design and Pharmacokinetic Characterization of Novel Antibody Formats for Ocular Therapeutics. Invest Ophthalmol Vis Sci. 2015;56(9):5390-400.

(2) Hutton-Smith LA, Gaffney EA, Byrne HM, Maini PK, Gadkar K, Mazer NA. Ocular Pharmacokinetics of Therapeutic Antibodies Given by Intravitreal Injection: Estimation of Retinal Permeabilities Using a 3-Compartment Semi-Mechanistic Model. Mol Pharm. 2017;14(8):2690-2696.

(3) Lamminsalo M, Taskinen E, Karvinen T, Subrizi A, Murtomäki L, Urtti A, Ranta VP. Extended Pharmacokinetic Model of the Rabbit Eye for Intravitreal and Intracameral Injections of Macromolecules: Quantitative Analysis of Anterior and Posterior Elimination Pathways. Pharm Res. 2018;35(8):153.

(4) del Amo EM, Rimpelä AK, Heikkinen E, Kari OK, Ramsay E, Lajunen T, Schmitt M, Pelkonen L, Bhattacharya M, Richardson D, Subrizi A, Turunen T, Reinisalo M, Itkonen J, Toropainen E, Casteleijn M, Kidron H, Antopolsky M, Vellonen KS, Ruponen M, Urtti A. Pharmacokinetic aspects of retinal drug delivery. Prog Retin Eye Res. 2017;57:134-185.

(5) Pitkänen L, Ranta VP, Moilanen H, Urtti A. Permeability of retinal pigment epithelium: effects of permeant molecular weight and lipophilicity. Invest Ophthalmol Vis Sci. 2005;46(2):641-6.
